# Electric powered wheelchair control using user-independent classification methods based on surface electromyography signals

**DOI:** 10.1007/s11517-023-02921-z

**Published:** 2023-09-26

**Authors:** Hassam Iqbal, Jinchuan Zheng, Rifai Chai, Sivachandran Chandrasekaran

**Affiliations:** 1 Department of Engineering Technologies, Swinburne University of Technology, John Street, 3122 Melbourne, Victoria Australia; 2 Department of Computing Technologies, Swinburne University of Technology, Melbourne, Australia

**Keywords:** Assistive technology, Machine learning, Electric powered wheelchair, Surface electromyography

## Abstract

Wheelchairs are one of the most popular assistive technology (AT) among individuals with motor impairments due to their comfort and mobility. People with finger problems may find it difficult to operate wheelchairs using the conventional joystick control method. Therefore, in this research study, a hand gesture-based control method is developed for operating an electric-powered wheelchair (EPW). This study selected a comfort-based hand position to determine the stop maneuver. An additional exploration was undertaken to investigate four gesture recognition methods: linear regression (LR), regularized linear regression (RLR), decision tree (DT), and multi-class support vector machine (MC-SVM). The first two methods, LR and RLR, have promising accuracy values of **94.85%** and **95.88%**, respectively, but each new user must be trained. To overcome this limitation, this study explored two user-independent classification methods: MC-SVM and DT. These methods effectively addressed the finger dependency issue and demonstrated remarkable success in recognizing gestures across different users. MC-SVM has about **99.05%** of both precision and accuracy, and the DT has about **97.77%** accuracy and precision. All six participants were successful in controlling the EPW without any collisions. According to the experimental results, the proposed approach has high accuracy and can address finger dependency issues.

## Introduction

There is a growing problem of aging in many countries, including Australia, China, and the USA [1]. The shortage of nursing resources for the elderly makes it difficult to meet the needs of disabled people. Fortunately, assistive technology (AT) is on the verge of solving this issue. Whenever an unfortunate event impairs a person’s ability to walk, AT becomes essential. A large contingent of people is physically disabled as a result of health problems or accidents. A smart assistive wheelchair can make a world of difference for people who have neck paralysis, quadriplegia, congenital gait disorders, or finger dependencies [2].

A wheelchair is a great AT for people with special needs. However, some wheelchair users with finger disabilities face dilemmas when operating conventional joystick control wheelchairs. Quadriplegics who lack control over their legs and arms are examples of this. As a result, they have trouble eating and going to the restroom every day. Furthermore, their fingers make it difficult for them to control traditional joystick wheelchairs [3]. By using hand gestures, the indirect control method operates the wheelchair. An accelerometer, a gyroscope, and a camera are usually used for recognizing hand gestures [4].

**Fig. 1:**
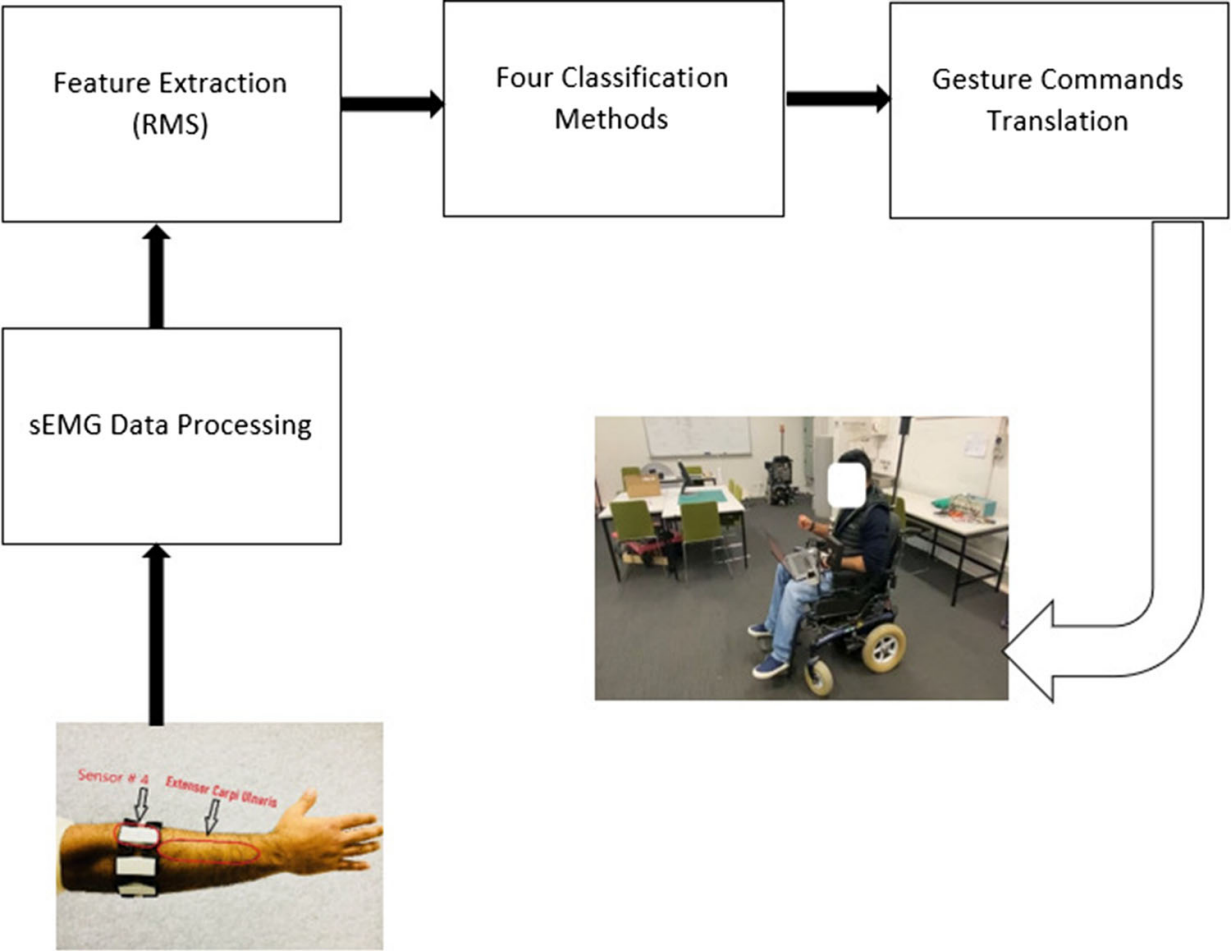
Graphical abstract

Several researchers have described methods for detecting and categorizing hand gestures in the literature. In general, these gestures can be divided into stationary gestures and dynamic gestures [5]. Systems based on centroid points are developed to control mouse cursors and video players [6]. Leap motion can be used to control robotic devices, simulate flights, and detect hand tremors [7]. Finger signs can also be recognized using orientation features in addition to head gestures [8]. An accelerometer-based neural network can be developed for high-accuracy hand gesture classification [9]. Hand gestures can be recognized using machine learning techniques as an alternative and user-friendly option. Controlling mobile robots or wheelchairs can be done through mild forced commonly used hand gestures.

The utilization of surface electromyography (sEMG) has become a prevalent technique for the recognition of hand gestures. The sEMG approach measures biosignal currents produced by motor units during muscle contraction [10]. The summation of motor unit action potentials is detected over the skin using sEMG. Due to its noninvasive and low-cost characteristics, sEMG-based gesture recognition systems are widely employed in human–machine interfaces, robot control, speech detection, and rehabilitation studies [11]. Amputees can also benefit from sEMG, as it allows them to control electrical powered wheelchair (EPW). Despite the benefits of sEMG-controlled prosthetics, a majority of amputees in the USA do not use this technology [12]. The low acceptance rate is typically attributed to the challenge of intuitive control over prostheses, which remains a significant hurdle for researchers to overcome [13].

This research study focuses on developing hand gesture recognition (HGR) methods to control EPW for individuals with disabilities, the elderly, and patients with multiple sclerosis. In literature, touch screens and graphical user interfaces have also been used to control wheelchairs. However, individuals with finger-related problems may still face difficulties while controlling the wheelchair effectively. To address this issue, this study employed an existing control system for an EPW that was originally controlled with a joystick.

**Fig. 2:**
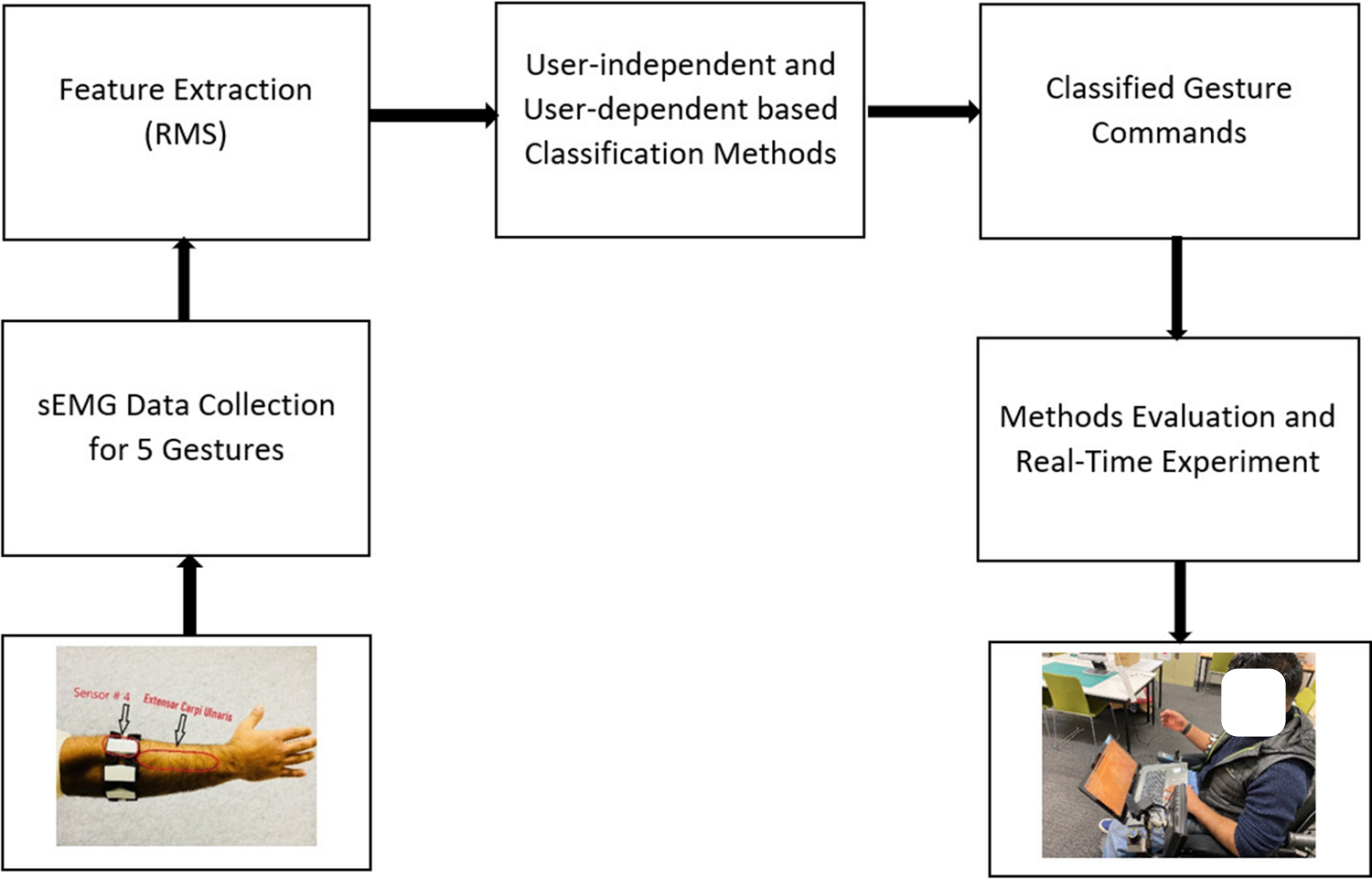
Steps in developing a hand gesture-based control for electric powered wheelchair system

This research study presents two user-dependent classification methods including linear regression (LR) and regularized linear regression (RLR) and two user-independent classification methods including multi-class support vector machine (MC-SVM) and decision tree (DT). Every time a new user is introduced, user-dependent classification methods need to be trained [14, 15]. To overcome this limitation, this study also investigates user-independent classification methods. Moreover, this study compares the performance of user-independent and user-dependent-based classification models.

This research study focuses on the evaluation of different algorithms using real-time sEMG data. In Section 2, the methodology is detailed, covering participant recruitment, hand gesture selection, training, and evaluation. Section 3 provides a description of the analysis conducted using the MC-SVM, DT, LR, and RLR methods, the graphical abstract is shown in Fig. 1.

## Methodology

This study conducted a research that entailed acquiring sEMG signals through the Myo armband and leveraged real-time sEMG data to assess both user-dependent and user-independent methods. Following this, this study applied preprocessing techniques to the sEMG signals and extracted the root mean square (RMS) feature. Subsequently, a model was trained using the RMS features, facilitating the accurate classification of sEMG signals.

### General structure and participant recruitment

The research study, approved by the Swinburne University of Technology Human Ethics Committee, employed the reliable and portable Myo armband for sEMG data acquisition, which has been extensively utilized for capturing sEMG signals. The general structure of this study is shown in Fig. 2. The primary data source consisted of sEMG signals collected through the Myo armband sensor. To ensure accurate analysis, a preprocessing step was applied, involving a data processing class implemented in MATLAB. The class effectively removed the DC offset and normalized the sEMG data using *z*-score normalization, standardizing the data for further analysis. Additionally, the class extracted the RMS feature from the normalized sEMG signals, providing valuable insights into the magnitude and intensity of muscle activities. This crucial information enabled the distinction between different muscle movements. The research study primarily focused on recognizing five hand gestures: fist, spread fingers, wave-in, wave-out, and rest gesture. The main objective was to develop a precise system capable of classifying and distinguishing these hand gestures based on sEMG signals. The study involved six healthy and able-bodied participants, all aged 18 years and above, with no history of neurological disorders or injuries to the shoulder, elbow, or wrist. Table 1 offers a concise summary of the participants’ demographic information, including age and height.

### Sensor placement protocol

**Fig. 3:**
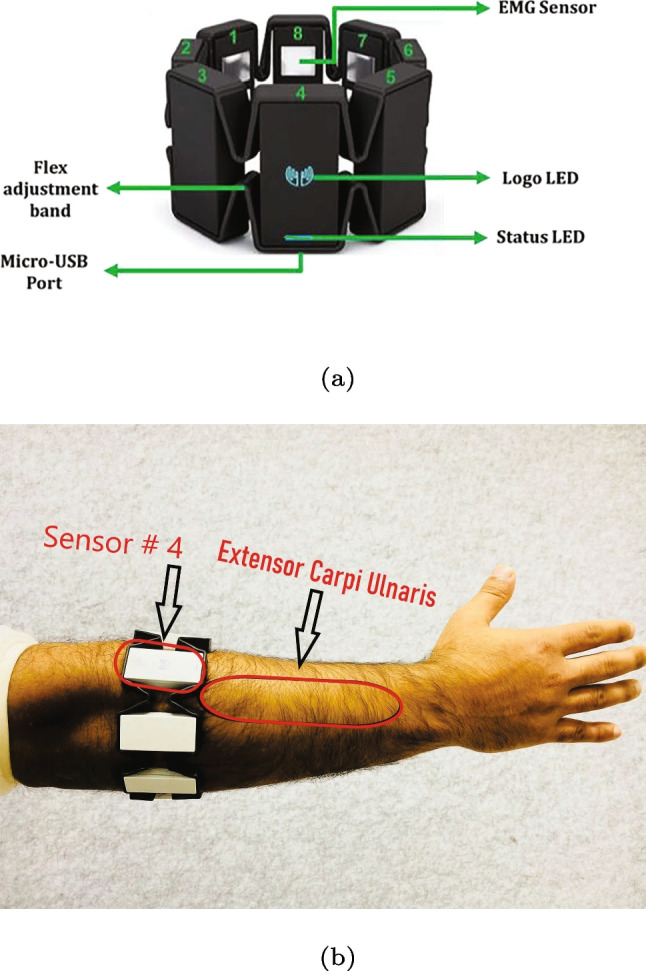
(a) Myo armband. (b) The Myo armband is placed on the dominant limb

The participants in the study were equipped with a Myo armband on their dominant limb, which included an inertial measurement unit (IMU) and eight dry electrodes, as shown in Fig. 3a. The Myo armband was carefully positioned approximately one inch distal to the elbow joint of the participant’s dominant limb, following the manufacturer’s guidelines. To ensure consistency and minimize variability between participants, the fourth sensor was specifically placed above the extensor carpi ulnaris muscle, as depicted in Fig. 3b. This deliberate positioning allowed for the precise placement of the IMU sensor on the dorsal side of the forearm in Sensor 4, in accordance with established recommendations [15].

### Gestures

**Fig. 4:**
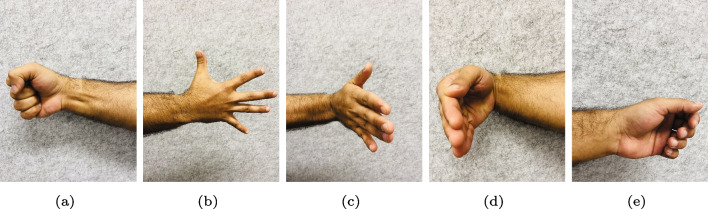
Five gesture library. **a** Fist gesture corresponds to a forward maneuver. **b** Spread fingers gesture corresponds reverse maneuver. **c** Wave-in gesture corresponds left maneuver. **d** Wave-out gesture corresponds right maneuver. **e** Rest gesture corresponds stop maneuver

During the experimental protocol, participants were instructed to execute five distinct hand gestures while wearing the Myo armband, as depicted in Fig. 4. Each gesture was performed in two iterations with moderate force and maintained for a duration of 5 s, followed by a two-second rest period. The sequence of gestures carried out by the participants followed the order of right, rest, left, rest, forward, rest, and reverse movements. To enhance the effectiveness of user-independent classification, each gesture was extended to a 5-s duration during the data collection process. This extension allowed for the collection of a larger number of data points for all gestures, except for the rest gesture, thereby optimizing the training time. As a result, each gesture was recorded for approximately 5 s, resulting in approximately 258 samples per gesture. However, for the rest gesture, data was collected only for a duration of 2 s, which consequently resulted in nearly half the number of data samples compared to other gestures.

**Table 1:** Participants data

Gender	Age (years)	Height (cm)
Male (5)	21$\pm 5$	172$\pm 6.5$
Female (1)	19	165

### Data collection

**Fig. 5:**
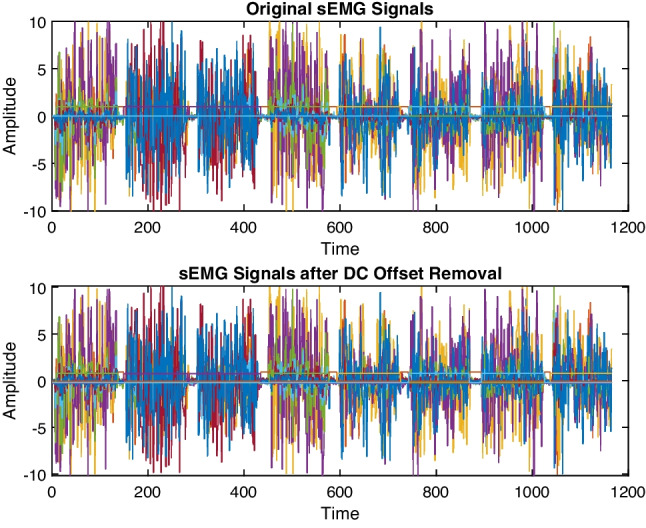
sEMG data collection with DC offset removal

The surface electromyography (sEMG) data were collected at a sampling frequency of 200 Hz. The recorded data was then transmitted to a computer via Bluetooth 4.0, where it was processed using a data acquisition graphical user interface (GUI) developed in MATLAB R2022b. The GUI was designed using the app designer toolbox and integrated with the Myo SDK MATLAB Mex wrapper toolbox [16]. In the preprocessing step for sEMG data, the DC offset was removed. The DC offset represents the average value of the signal, which can vary due to factors like myo armband placement and skin impedance. Removing the DC offset is essential to ensure that the EMG signal is centered around zero and to eliminate any potential drift in the signal over time. This step helps in enhancing the accuracy of the subsequent analysis and classification of the sEMG data. Figure 5 demonstrates the effect of DC offset removal on the sEMG signal.

### Feature extraction

**Fig. 6:**
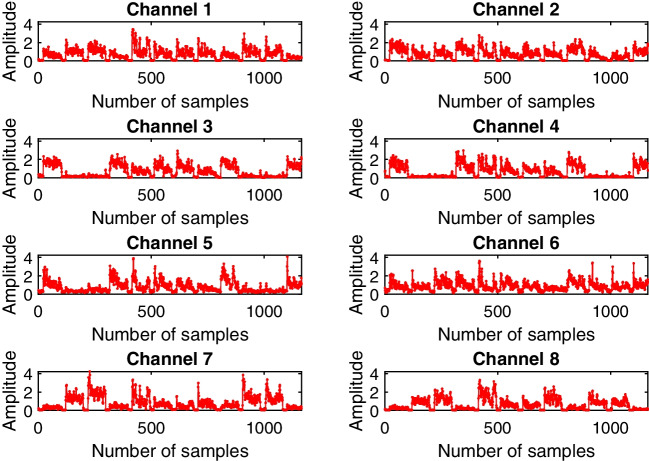
sEMG data was collected from each channel of the Myo armband

sEMG signals are non-stationary, and therefore require conversion into a low-dimension feature set, as illustrated in Fig. 6. These features are derived from subsets of the raw signals referred to as windows. The processing time is influenced by the window’s length, and a longer window may result in a longer delay between signal generation and prosthesis actuation. In practice, a delay between 100 and 259 ms is often chosen to maintain the total delay under 300 ms. An RMS feature set has been extracted from the raw sEMG signals with over-lapping signal windows of 200 ms, which is updated every 40 ms.

It will be important to note that as the window moves forward in time, it may overlap or disjoint according to the gap between consecutive windows. Overlapping windows are those with intervals shorter than the window’s length, while disjoint windows are those with intervals equal to the window’s length.

**Fig. 7:**
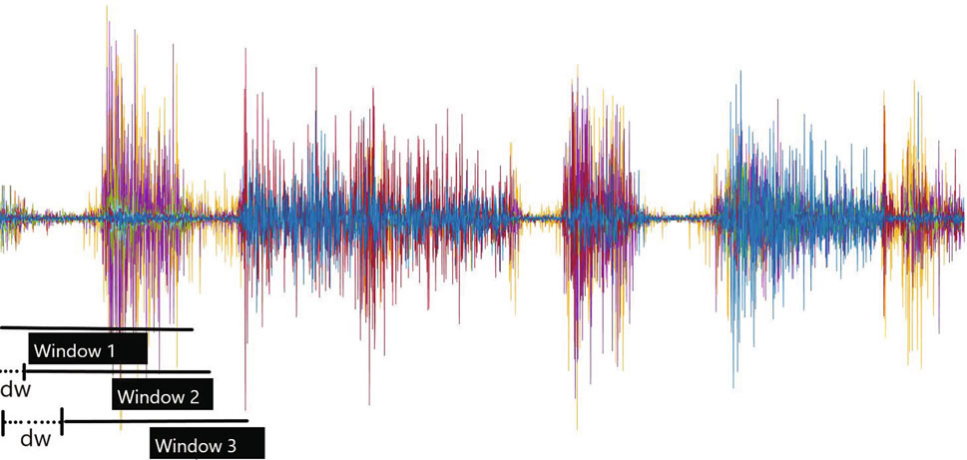
Overlapping features are extracted from the raw sEMG signal

A representation of the overlapping window can be seen in Fig. 7. A disjoint window causes a longer delay, so the overlapping window is often preferred. To extract a feature set, various features are available for each window, including the time domain, frequency domain, and time-scale domain. Power spectrum parameters are commonly used to study muscle exhaustion, but they may not offer enough information about signals with non-stationary or transitory properties in the time domain. In contrast, wavelet analysis provides a time-domain feature that incorporates both frequency and time information, even though it has low computational efficiency. Despite the lack of frequency information, time-domain features are most widely used in myoelectric control due to their quick processing time and high computational efficiency. The RMS of an sEMG signal is an example of a time-domain feature. A comprehensive review can be found at [17]; the RMS is explained below:

#### Root mean square

The square root of the mean value of a squared function is the RMS value. RMS is one of the technologies commonly used to evaluate sEMG. The formula is as follows:

1 $$\begin{aligned} x_{RMS} = \sqrt{\frac{(x_1^2+x_2^2+...x_n^2)}{n}} \end{aligned}$$

where $x_1 + x_2+...x_n$ are the data points and *n* is the samples of the sEMG data.

### Study design

**Fig. 8:**
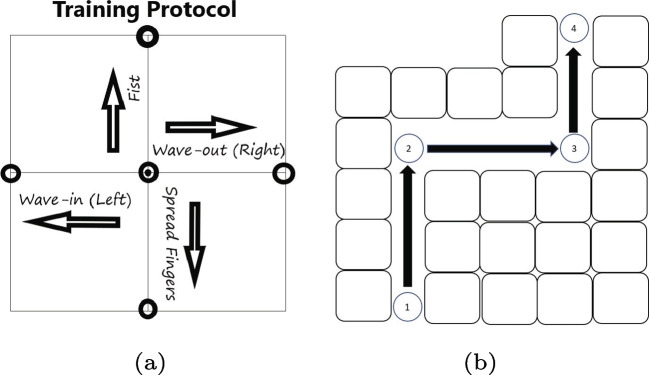
(a) Participants received visual feedback indicating their estimated position by a circle at the origin. During a training session, the four targets (right, left, forward, and reverse) were used. (b) The layout of the trajectory of the implementation wheelchair control system using hand gestures

In this research study, participants were seated in front of a computer that was attached to the powered wheelchair while wearing the Myo Armband on their dominant limb. To indicate the origin, participants were asked to adopt a natural posture. Participants were instructed to control the horizontal and vertical velocity of the cursor using the fist and spread fingers gesture and the wave in and wave out gesture, respectively, as depicted in Fig. 8a. The trajectory layout is presented in Fig. 8b, and all participants were required to navigate the wheelchair from point 1 to point 4 according to the trajectory layout.

### Training session

After wearing the Myo armband, participants were instructed to perform a series of five specific gestures. These gestures, commonly used in daily tasks, were categorized using four machine learning methods: two user-dependent algorithms based on LR and RLR and two user-independent algorithms based on DT and MC-SVM. Each trial involved participants executing the five gestures with moderate force. Two repetitions of each gesture were performed with mild force, with a two-second pause between each repetition. To capture as much data as possible, the gestures were held for 5 s during data segmentation.

**Fig. 9:**
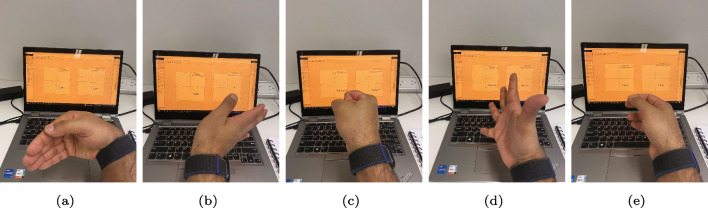
Real-time training for (a) left gesture, (b) right gesture, (c) fist gesture, (d) spread fingers gesture, and (e) rest gesture

The training consisted of participants following the target in two DOFs cartesian coordinates using hand gestures in four directions displayed one at a time on the screen as shown in Fig. 9. The end-point is reached in 5 s, stayed at for 2 s, and then returned to the origin in 5 s. In this research study, the vertical position of the target was controlled by wrist flexion and extension, while the horizontal position was controlled by the fist and spread fingers gestures. Participants were required to maintain the gestures until the cursor returned to its origin after the researcher had described the correlation between each gesture and the degree of freedom. In contrast to traditional approaches, this research study used a training method where the time and memory allocation for each target were pre-determined. Based on the real-time learning performance of each participant, this research study determined the amount of time and memory allocated to each target Table 2.

**Table 2:** Table of Performance Metrics during training

Number of missed targets	Missed targets when compared to 5 total targets
Completion rate	Ratio of completed to total targets
Completion time	Time taken to complete a target

### Evaluation

The evaluation process in this study was divided into two sections: a training section and a testing section. The training section was devoted to the evaluation of training methods, while the testing section focused on measuring the performance of machine learning models in terms of accuracy, precision, recall, and F1 score. During sEMG data collection, five targets were presented individually, and participants’ performance was evaluated using performance metrics as presented in Table 3. If a target is incomplete, it is counted as a missing target. Completing a target requires the cursor to be moved to the target and held there until the cursor returns to the original target within 5 s. If the time exceeds 5 s, the target is identified as missed. The completion ratio is calculated by dividing the number of reached targets by the total number of targets. After completing a target, the cursor must return to the origin before proceeding to the next target. Each target’s completion time and the time taken to complete it are recorded. All six participants successfully hit the 5 targets within 112 s, which was the designated completion time according to the training protocol. As a result, the completion ratio for all participants was $100\%$, indicating that each participant achieved the goal of hitting all targets within the specified time frame during training. This study evaluated each participant once.

### Performance metrics for user-independent classification methods

An accuracy performance metric is defined as the ratio of correctly predicted observations to total observations. In machine learning (ML), accuracy is a metric that measures the overall correctness of a model’s predictions. It is defined as the ratio of the number of correct predictions to the total number of predictions made by the model. Accuracy is calculated using the following formula in Eq. (2). ML classification models are commonly assessed by accuracy metrics, where TP is true positive, TN is true negative, FP is false positive, and FN is false negative [18].

2 $$\begin{aligned} Accuracy = \frac{TP+TN}{TP+TN+FP+FN} \end{aligned}$$

#### Precision

In ML, precision is a metric that measures the accuracy of positive predictions made by a model. It is defined as the ratio of true positive predictions to the total number of positive predictions made by the model. Precision is calculated using the following formula in Eq. (3):

3 $$\begin{aligned} Precision =\frac{TP}{TP+FP} \end{aligned}$$

#### Recall sensitivity

In ML, recall is a metric that measures the ability of a model to correctly identify positive instances from a dataset. It is defined as the ratio of true positive predictions to the total number of positive instances in the dataset. A recall is calculated using the following formula in Eq. (4):

4 $$\begin{aligned} Recall =\frac{TP}{TP+FN} \end{aligned}$$

#### F1 score

F1 score is a metric that combines precision and recall into a single score. It is a measure of a model’s accuracy that takes into account both the number of true positive predictions and the number of false positive and false negative predictions. The F1 score is calculated as the harmonic mean of precision and recall as shown in Eq. (5). The F1 score ranges from 0 to 1, with a higher score indicating better performance. A perfect F1 score of 1 indicates that the model has achieved both high precision and high recall. The F1 score is commonly used as a performance metric for classification tasks, especially in cases where the dataset is imbalanced or the cost of false positives and false negatives is different. By taking into account both precision and recall, the F1 score provides a more balanced evaluation of an ML model’s performance than accuracy alone.

5 $$\begin{aligned} F1 score = \frac{2TP}{2TP+FP+FN} \end{aligned}$$

**Table 3:** Comparison between proposed and state-of-the-art-work in literature

Classification model	Number of gestures	Recognition accuracy
Convolutional neural network [22]	6	90%
Feed-forward ANN classifier [23]	5	92.45%
Dynamic time warping and affinity propagation [24]	18	94.60%
Adaptive least square SVM [25]	7	92.90%
Proposed linear regression	5	94.85%
Proposed regularized linear regression	5	**95.88%**
Proposed decision tree	5	97.77%
Proposed MC-support vector machine	5	**99.05%**

### Performance metrics for user-dependent classification methods

The following metrics are widely used to assess the performance of a regression model *f*. The sum of squares is shown below:

6 $$\begin{aligned} SS_{tot} = \sum _{i-1}^{m} (y_i - \bar{y})^2 \end{aligned}$$

The second is the residual sum of squares:

7 $$\begin{aligned} SS_{res} = \sum _{i-1}^{m}( (y_i)- f(x_i))^2 \end{aligned}$$

The hyperparameter optimizations were performed based on 10-fold cross-validation. Randomly selected data samples were used for evenly distributed training samples in each fold. $R^2$ was used as a performance metric, which is a common regression metric. It is defined as follows:

8 $$\begin{aligned} R^2 = 1- \frac{SS_{res}}{SS_{tot}} \end{aligned}$$

The second term in Eq. (8) is the variance of the labelled data divided by the mean square error. A value of $R^2=1$ is at its maximum, and the closer to it, the higher the performance. If the mean square error is greater than the variance of labels, $R^2$ could be negative, which represents poor model performance. The hyperparameter value that resulted in the highest cross-validation $R^2$ was selected during a hyperparameter optimization.

## Classification methods

To classify the data sets obtained during training, two user-independent and two user-dependent classification methods were implemented. More specifically, Sections  3.1 and 3.2 discussed the multi-class support vector machine (SVM) and the decision tree (DT) classification methods. A linear regression (LR) method and a regularized linear regression (RLR) method were also used as the third and fourth classification methods. All these methods have been used to classify five hand gestures. This study employed user-dependent classification models, LR and RLR, which are specifically tailored to individual users. These models do not depend on data from other users, making them effective for recognizing gestures for a single participant at a time. However, when introducing a new user, these models require training with data from that particular user to ensure accurate gesture recognition. In order to overcome the user-dependent nature, this study further investigated two user-independent ML models including MU-SVM and DT. Following is an explanation of how these classification methods are implemented.

### User-independent multi-class support vector machine method

Support vector machines (SVM) are widely used for classification tasks in supervised machine learning. By finding the hyperplane that maximizes the margin between the two classes, it divides the data into two classes. However, the SVM algorithm needs to be modified for multi-class classification problems. The one-vs-all (OvA) method, also called one-vs-rest, is one approach to solving multi-class classification problems using SVM.

Multiple binary classifiers are trained using the OvA method, each responsible for differentiating one class from the rest. Based on the predicted score, the highest class is selected for the final prediction. One-vs-one (OvO) is another approach to solving multi-class classification problems using SVM. Using this method, multiple binary classifiers are trained, each of which separates two classes. The final prediction is based on a voting scheme, where each binary classifier votes for one class. OvA approach for hand gesture recognition is the main focus of this study.

Both OvA and OvO have advantages and disadvantages. In comparison to OvO, OvA is computationally more efficient and requires fewer classifiers to be trained, but it can be less accurate. OvO, on the other hand, trains more classifiers, making it computationally more expensive, but it can result in better accuracy since it takes all classes into account. Multi-class SVM is a learning method in which linear functions are mapped into high-dimensional feature spaces instead of modelling probabilities through training data. A support vector kernel is used to map the input data to a high-dimensional feature space, allowing the problem to be processed linearly. In the optimization process, support vectors are samples whose multipliers are not zero.

In SVMs, the global minimum is always found since the objective is to decrease the bound on the structural risk rather than the empirical risk. For MC-SVM-based gesture recognition, the SVM should be extended to a k-class problem as it is a binary classifier. This study adopted the one-vs-all strategy, which is a pairwise approach and needs to train $k(k-\frac{1}{2})$ SVM classifiers. Matlab classification learner includes many classification algorithms. This research tested each of these classifiers during non-real-time classification. In this study, SVM algorithms were emphasized since they achieved the highest accuracy as compared to decision trees, linear regression, and regularized linear regression algorithms.

A support vectors (SVs) kernel is used to map the data from the input space to a high-dimensional feature space, which facilitates the linear processing of the problem. At the end of optimization, SVs have multipliers that are not zero. In order to achieve this goal, SVMs minimize the following Lagrange formulation, Lagrange is a function of the model parameters *W* weight vector and *b* bias term as well as Lagrange multipliers $\alpha$ as shown as in (9), where the first term is a regularization term that encourages the weights to be small, second is the margin term that measures the quality of the classification margin, and third term $\alpha _i$ is a constraint term enforces the constraint that the Lagrange multiplier is non-negative.

9 $$\begin{aligned} L_{p}\equiv {1\over 2}\Vert w\Vert ^{2}-\sum \limits _{i=1}^{l}\alpha _{i}y_{i}(x_{i}w+b)+\sum \limits _{i=1}^{l}\alpha _{i} \end{aligned}$$

10 $$\begin{aligned} f(x)=\sum \limits _{i=1}^{N}\alpha _{i}y_{i}k(x, x_{i})+b \end{aligned}$$

The decision function can be seen in (10), where *k* represents the kernel function, and this study has used the linear kernel function in this gesture recognition problem, $x_i$ represents the training samples, $y_i$ represents their class labels, and *b* represents the model parameters. Using SVM information for binary classifiers, one solution for k-class pattern classification is to extend it to k-class pattern classification. Multiple pattern recognition problems can be classified and identified using SVMs with supervised learning classifiers. SVMs with multiple classes can be used to identify gestures based on their trajectory. The high-dimensional data is separated by SVM so that errors are reduced since it is linearly incomparable data. A one-vs-all approach is used for gesture recognition in this paper. This method uses k SVMs, each of which separates one class from all other classes in the training set [19].

### User independent decision tree method

A decision tree consists of a root node, internal nodes, and leaf nodes, where leaf nodes represent classes, and non-leaf nodes indicate attributes of classes. In the root node, sample data, including values for different attributes, are placed. As a result of the rules in non-leaf nodes, the decision tree splits values into multiple branches corresponding to different attributes. Finally, leaf nodes assign which class input data belong to. It is easy to understand and interpret decision trees. Multi-feature pattern classification is well suited to their ability to fusion diverse information. Additionally, they reduce the search range between classes for classification by using their sequential structure of branches. A decision tree can perform well on large data in a short amount of time, which is a significant advantage for implementing real-time classification systems [20].

The decision tree can be used to predict responses to data, also called a classification tree or regression tree. Trees begin at the root and are divided into leaf nodes as they progress down the tree. During response prediction, the decision starts at the beginning node and moves down to the leaf node. The leaf node then stores the response. Classification trees provide output in the form of true or false, whereas regression trees give numerical results. As compared to other classifiers, the training and testing accuracy obtained in this case is lower than multi-class SVM. DT has the highest accuracy of $97.77\%$ among 6 different participants.

### User dependent regression methods

Supervised learning, a branch of machine learning, involves the use of algorithms such as linear regression to model the relationship between two variables. Linear regression specifically utilizes a linear equation to fit observed data, with one variable acting as the independent variable and the other as the dependent variable. The aim of linear regression is to predict the dependent variable, denoted as *y*, from the independent variable, denoted as *x*. To accomplish this, the algorithm seeks to establish a linear relationship between input *x* and output *y*, and ultimately utilize the best-fitting line to predict the continuous variable outcome. The best-fitting line represents the relationship between the independent variable *x* and the dependent variable, and the algorithm endeavors to minimize the sum of the squared differences between the data points and the regression line to obtain the optimal fit.

11 $$\begin{aligned} y_i = \beta _0 + \beta _1 x_{i_1} + ...+\beta _p x{i_p} +\epsilon _i y_i = x_i^T\beta + \epsilon _i \end{aligned}$$

Noting that *T* denotes the transpose operator, the hypothesis function for linear regression is given by $x_i\beta$, where $x_i$ and $\beta$ represent vectors, and $x_i\beta$ represents the inner product between the two vectors.

12 $$\begin{aligned} y=\theta _1 + \theta _2x \end{aligned}$$

In the context of linear regression, where *x* denotes the input training data and *y* represents the corresponding labels, the cost function *J* is defined as the root mean squared error (RMSE) between the predicted *y* value and the true *y* value.

13 $$\begin{aligned} J= \frac{1}{n} \sum _{i=1}^n (pred_i - y_i)^2 \end{aligned}$$

**Fig. 10:**
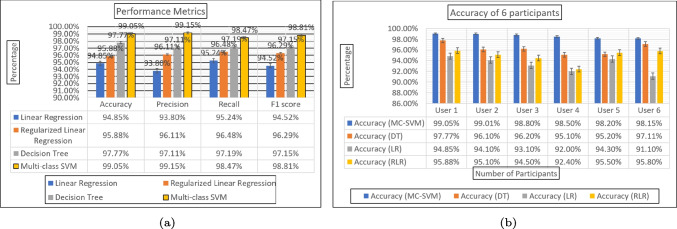
(a) Performance metrics for all the algorithms. (b) Accuracy of all the ML-Models among the 6 able-bodied participants

In order to achieve the best-fit line and minimize the cost function (i.e., minimize RMSE) in linear regression, the model employs gradient descent to update the values of $\theta _1$ and $\theta _2$ iteratively. The initial values of $\theta _1$ and $\theta _2$ are set randomly and are then updated iteratively until the minimum cost function is reached. Specifically, the feature set is represented as ${\textbf {X}} \in \mathbb {R}^{P\times n}$, where *P* is the dimension of the feature and *n* is the number of samples, and the labels are represented as ${\textbf {Y}} \in \mathbb {R}^{n \times Q}$, where *Q* is the dimension of the output target. The ultimate objective is to estimate $\hat{y}_{n+1}$, which represents the prediction for a new observation $x_{n+1}$.

#### Linear regression

The linear regression assumes a linear relationship between *X* and *Y* as follows:

14 $$\begin{aligned} \hat{Y} = X^TW \end{aligned}$$

where $W \epsilon R^{P \times Q}$ is a weight matrix. The cost function of least squares in $q^{th}$ DOF out of *Q* DOFs is

15 $$\begin{aligned} J_q = \sum _{t=1}^n |e_q(t) |^2 \end{aligned}$$

where *e* is the error term defined as $e=Y-X^TW$. The weight matrix that minimizes (14) is shown below:

16 $$\begin{aligned} W= (XX^T)^{-1} XY \end{aligned}$$

#### Regularized linear regression

Regularized linear regression has the same linear model as (15) with an additional term in the cost function. The cost function is given as follows:

17 $$\begin{aligned} J_q = \sum _{t=1}^n |e_q(t) |^2 + \lambda W_q^T W_q \end{aligned}$$

where the additional term is the $l_2$ regularization term with the positive constant $\lambda$. $l_2$ normalization is a computational way to avoid overfitting and instigate its general ability. The weight matrix that minimizes (16) is

18 $$\begin{aligned} W= (XX^T+\lambda I)^{-1} XY \end{aligned}$$

where *I* is an identity matrix. The regularization constant $\lambda$ is selected within a logarithmically spaced vector $[10^{-3},....,10^{3}]$ by a grid search based on a *k*-fold cross-validation accuracy.

## Results

The evaluation of diverse machine learning techniques for the classification of sEMG data gathered from the library of five gestures through the utilization of the Myo armband sensor involved assessing their accuracy. Additionally, precision, recall, and F1 score were computed to gain deeper insights into the models’ performance. Precision gauges the model’s capacity to exclusively yield relevant data or accurately identify gestures associated with a specific class. On the contrary, recall quantifies the ratio of true positive classifications (accurately classified samples) to false negatives (samples mistakenly classified as a different class). The F1 score represents a measure of the harmonic mean between precision and recall, offering a balanced perspective on the model’s precision and recall performance [18].

**Fig. 11:**
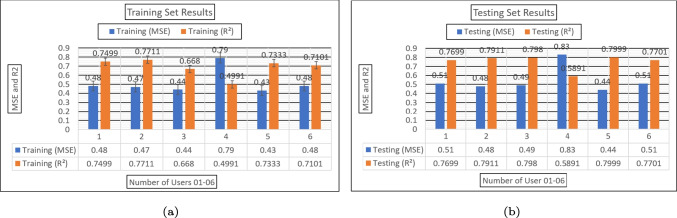
(a) Training MSE and $R^2$ of RLR ML-Model among the 6 able-bodied participants. (b) Testing MSE and $R^2$ of RLR ML-Model among the 6 able-bodied participants

The MC-SVM, DT, LR, and RLR achieved accuracy rates of $99.05\%$, $97.77\%$, $95.88\%$, and $94.85\%$ respectively. Assessment was based on criteria including accuracy and the confusion matrix. Figure 10 provides insights into the accuracy, precision, recall, and F1 score across all methods. Notably, when utilizing the MC-SVM, the overall  precision reached $99.15\%$, with recall at $98.47\%$, and an F1 score of $98.81\%$ significantly outperforming other algorithms scrutinized in this research study. A graphical representation of the mean squared error and coefficient of determination for user-dependent methods in both training and testing can be found in Fig. 11.

**Fig. 12:**
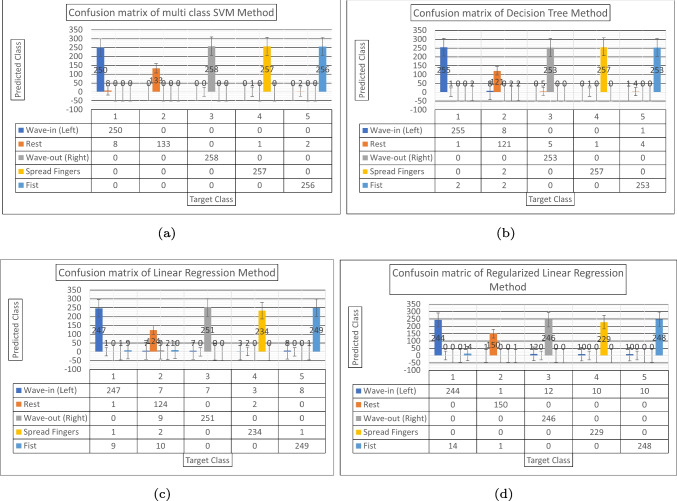
(a) Confusion matrix of Multi-class SVM method. (b) Confusion matrix of decision tree method. (c) Confusion matrix of linear regression method. (d) Confusion matrix of regularized linear regression method

**Fig. 13:**
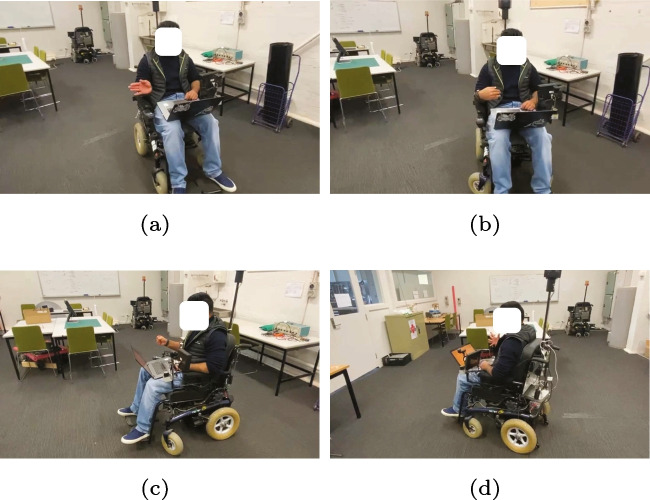
(a) Wave-out gesture for right maneuvers. (b) Wave-in gesture for left maneuvers. (c) Fist gesture for forward maneuvers. (d) Spread fingers gesture for reverse maneuvers

Multi-class SVM achieved the highest accuracy of $99.05\%$ which is also higher when compared with other existing studies. In this work, a comparison has been made between MC-SVM, DT, LR, and RLR, all of these methods applied to control an electric-powered wheelchair. The confusion matrix for each of the algorithms analyzed in this study can be found in Fig. 12, and real-time implementation of all the algorithms on a wheelchair is shown in Fig. 13. Upon the introduction of a new user for wheelchair control, the LR and RLR user-dependent models require training. On the other hand, the user-independent models, DT and MC-SVM, eliminate the need for training with new users. A new user simply needs to calibrate the Myo armband and navigate the powered wheelchair.

Table 3 provides a comparison of various classification models used for gesture recognition, including convolutional neural network (CNN) [22], feed-forward ANN classifier [23], dynamic time warping and affinity propagation [24], and adaptive least square SVM [25] methods, and the CNN model achieved a recognition accuracy of $90\%$ for 6 gestures. The feed-forward ANN classifier achieved a slightly higher accuracy of $92.45\%$ for 5 gestures. Dynamic time warping and affinity propagation achieved a recognition accuracy of $94.60\%$ for a larger set of 18 gestures. The adaptive least square SVM achieved an accuracy of $92.90\%$ for 7 gestures. This research study proposed four methods for hand gesture recognition. The proposed LR model achieved an accuracy of $94.85\%$ for 5 gestures. The proposed RLR improved the accuracy further to $95.88\%$ for the same set of gestures. The proposed DT model achieved an accuracy of $97.77\%$. Nevertheless, the MC-SVM model attained the peak accuracy of $99.05\%$ for five hand gestures. Comparing the outcomes of this research study with those from the studies outlined in Table 3, the proposed MC-SVM model showcases superior performance over other models, including CNN, ANN, DTW, and least square SVM methods, in terms of recognition accuracy. This underscores the effectiveness and exceptional capabilities of the presented model for gesture recognition tasks, even with a smaller set of gestures. In summary, this study demonstrates the successful application of the proposed MC-SVM model, achieving an impressive recognition accuracy of 99.05% for gesture classification. This accomplishment surpasses the performance of existing models and underscores its potential for practical deployment in real-world gesture recognition systems.

To conclude, the machine learning models employed in this study successfully classified five distinct gestures: fist, wave-in (left), wave-out (right), spread-fingers, and hand at rest. Notably, the MC-SVM model exhibited enhanced accuracy in comparison to other models investigated in this study. The study conducted real-time experimental validation using an EPW, yielding promising outcomes. By harnessing sEMG data and the RMS feature, a confusion matrix was derived for the complete dataset, encompassing approximately 258 samples for each gesture, except for the rest gesture, which had half the sample count. This discrepancy originates from the experimental protocol, wherein the rest gesture was recorded for 2 s, whereas other gestures were captured for 5 s, contributing to the observed difference in sample quantities.

## Discussion

### Linear regression and regularized linear regression methods

Using linear regression (LR) and regularized linear regression (RLR), the results showed that it is possible to achieve recognition accuracy of up to $95.88\%$ for the six subjects when using sEMG data to classify five hand gestures. This study trained and tested two different ML models. The RLR model achieved a recognition accuracy of $95.88\%$, higher than the LR model’s $94.85\%$. As a result, RLR produced more accurate results when dealing with the complexity of the hand gesture recognition task.

Overfitting could be one reason for the worse performance of LR. Essentially, overfitting occurs when a model fits the training data too well, resulting in poor generalization to new data once the model has been trained. By adding a regularized term $\lambda$ to the cost function, RLR reduces the complexity of the model. By doing so, the model can avoid overfitting and produce more accurate results. Regularized regression could also improve performance because it effectively addresses multi-collinearity. Multi-collinearity occurs when two or more predictors are highly correlated in regression analysis. As a result, regression coefficient estimates can be unstable and inconsistent. This issue can be mitigated by regularizing regression to reduce the magnitude of coefficients for highly correlated predictors. This study concluded that RLR is a promising approach for hand gesture recognition with high accuracy and low mean squared error, indicating that this approach can handle the complexity of the task and produce more accurate results. The potential for RLR in other applications in this field needs to be explored further. Whenever a new user is introduced, it becomes essential to train these models. In the upcoming section, this study will explore two algorithms that are independent of user-specific training.

### Decision tree method

A decision tree (DT) classifier, a widely used ML algorithm for multi-class classification issues, was implemented to identify five hand gestures. Study objectives included evaluating the performance of the decision tree for recognizing hand gestures for controlling the powered wheelchair’s movement. Model accuracy, precision, recall, and F1 score were evaluated using real-time sEMG data, which was used to train and test the model. According to the results, the decision tree achieved an accuracy of 97.77% and a precision of 97.11%. The results show that the model recognizes hand gestures for controlling the powered wheelchair very effectively. Additionally, six able-bodied participants were used in a real-time application test using an EPW. Based on a set of hand gestures performed by the six participants, the DT was able to recognize the gestures accurately in real time. As a result, the model is effective in practical applications, such as controlling the movement of PW. In real-world applications, a DT model is an attractive option due to its simplicity and computational efficiency. It is possible to use this model in practical applications for hand gesture recognition in powered mobility devices because of its high accuracy and precision.

Based on DT results, it appears that the decision tree classifier captured the relationships between features and classes effectively, resulting in accurate predictions. It is important to note that decision trees are prone to overfitting. In other words, they may fit the training data too closely, resulting in poor generalization performance. There are several ways to address this issue, including pruning, assembling, or using a random forest classifier, which is an extension of a decision tree. A decision tree classifier can be a useful tool for solving multi-class classification problems, and it was able to effectively capture data relationships with an accuracy rate of 97.77%. The risk of overfitting must, however, be considered and techniques employed to prevent it. The performance of sEMG data-based gesture recognition was improved after classification, but the significant drawback was the low recognition accuracy observed after the classification of the data. To improve the accuracy, the next section will study MC-SVM method.

### Multi-class support vector machine method

The outcome of utilizing the Myo armband muscle sensor in the MC-SVM classification process shows not only an improvement in performance but also the capacity of the model to identify each gesture individually. Additionally, the MC-SVM technique is user-independent, meaning it doesn’t require any adjustment or training when a new user is introduced. The objectives included evaluating the performance of the MC-SVM model in recognizing hand gestures for controlling EPW. Training and testing were conducted on real-time sEMG data of hand gestures, and accuracy, precision, recall, and F1 score were used to assess performance. Results showed that the multiclass SVM achieved a precision of $99.15\%$ and an accuracy of $99.05\%$. According to these results, the model was highly effective at recognizing hand gestures.

In order to further validate the model, six able-bodied participants and a powered wheelchair were used in real-time application tests. Participants performed a set of hand gestures, and the multi-class SVM accurately recognized the gestures in real time. Consequently, the model can be utilized in practical applications, such as controlling the movement of an electric-powered wheelchair. This study has demonstrated that the MC-SVM is effective for recognizing hand gestures in an electric-powered wheelchair. The high accuracy and precision achieved during training and testing, as well as the successful real-time application, support the potential of this model for practical applications for controlling powered mobility devices.

## Conclusions and future work

As a result of this research, the following contributions were made. With an overall average accuracy of $98.61\%$ for 6 different able-bodied users, the proposed MC-SVM machine learning model demonstrated a remarkable response time of less than 1 s for hand gesture commands, processing, and execution. Additionally, this study achieved the best recognition accuracy of approximately $99.05\%$. The user-independent MC-SVM-based ML classification model enables new users to control the EPW without the need for re-training. It has been experimentally demonstrated that an electric-powered wheelchair can be controlled by users to complete all possible movements efficiently. This study aimed to enhance the accuracy of hand gesture recognition using sEMG data by developing four classification models. Two models were user-independent, and two were user-dependent. The study compares the different models and aims to determine which of them is most suitable for use in real-time applications by comparing the different models. The goal was to enhance the interaction between humans and computers through improved hand gesture recognition. The study utilized LR, RLR, DT, and MC-SVM approaches. The most accurate results were achieved using a MC-SVM with an accuracy of 99.05%. The DT method also had favorable results with an accuracy of 97.77%. The proposed system was found to be comfortable to use based on six participants. Future work will include comparisons with advanced deep learning-based gesture recognition techniques and an analysis of subject variability among more users, and recruit non-able-bodied participants to validate the experiment. The study also demonstrated the viability of the proposed system for human–machine interaction through its successful application to control an electric-powered wheelchair.
